# Consultation Lag and Early Improvement in Pediatric Daily Headache: A Retrospective Observational Study

**DOI:** 10.3390/medsci14040441

**Published:** 2026-07-27

**Authors:** Sachi Tokunaga, Hideki Shimomura, Eisuke Terasaki, Naoko Taniguchi, Saeka Yoshitake, Ayuko Usami, Shintaro Iwamoto, Yasuhiro Takeshima

**Affiliations:** 1 Department of Pediatrics, School of Medicine, Hyogo Medical University, Nishinomiya 663-8501, Japan; sa-tokunaga@hyo-med.ac.jp (S.T.);; 2 Department of Pediatrics, Kawanishi City Medical Center, Kawanishi 666-0017, Japan

**Keywords:** pediatric headache, daily headache, consultation lag, referral timing, clinical outcomes, prognostic factors

## Abstract

Background: Pediatric daily headaches are refractory and disabling. However, the influence of the interval between daily headache onset and initial specialist consultation on clinical outcomes remains unknown. Methods: This retrospective observational study included 51 children and adolescents presenting with daily headaches. Consultation lag was defined as the interval between the date when the headache first became daily and the initial consultation. The primary outcome was headache improvement (≥50% reduction in monthly headache days) at 3–4 months, and the secondary outcome was improvement at 6–7 months. Multivariate logistic regression and receiver operating characteristic curve analyses were performed. Results: Improvements were observed in 41.2% of patients at 3–4 months and 60.0% at 6–7 months. Each additional week of consultation lag was associated with a 12% decrease in the odds of a 3–4-month improvement (adjusted odds ratio, 0.875; 95% confidence interval [CI], 0.783–0.979; *p* = 0.020). Receiver operating characteristic curve analysis identified an exploratory 5-week threshold (area under the curve, 0.837; 95% CI, 0.711–0.963), with markedly lower odds of early improvement when exceeding this threshold in this cohort (adjusted odds ratio, 0.040; 95% CI, 0.007–0.238; *p* < 0.001). Comorbid postural orthostatic tachycardia syndrome was associated with the absence of 3–4-month improvement (*p* = 0.003), whereas other comorbidities were not. Conclusions: A shorter consultation lag was independently associated with early improvement in pediatric daily headaches regardless of comorbidities. Patients presenting within this exploratory 5-week window were substantially more likely to improve early in this cohort; however, this threshold requires prospective external validation before it can inform clinical decision-making. These findings support consideration of prompt specialist referral following the onset of daily headache in children.

## 1. Introduction

Headache is one of the most common complaints in the pediatric population and represents a significant clinical concern. Headaches not only cause physical discomfort in children but also substantially affect their quality of life, including school attendance, academic performance, and psychosocial functioning [1]. Migraine, in particular, imposes a considerable burden on children and adolescents. Further, as the headache frequency increases, limitations in daily activities increase markedly, and school functioning is severely disrupted [2]. Frequent primary headaches also show a high comorbidity with learning difficulties, potentially related to prefrontal cortical dysfunction or psychological factors, such as fear of failure [3]. This burden extends beyond the child to the family, contributing to parental work absenteeism and further affecting the child’s overall quality of life. Taken together, these compounding effects highlight the urgent need for comprehensive clinical evaluation, robust longitudinal research, and targeted educational and healthcare policies [2,3] and underscore the importance of intervention before frequent headache escalates into more refractory patterns.

When primary headaches increase in frequency and occur daily or near daily, they pose major therapeutic challenges in pediatric neurology. Within the current international classification, the most severe of these high-frequency states is defined as chronic migraine (CM). A large body of literature has reported these cases as chronic daily headache (CDH), a frequency-based clinical concept defined by Silberstein that predates CM. Despite its widespread historical use, CDH is not formally included in official classifications [4,5]. The prevalence of CDH has been reported to be 1.68% among children aged 5–12 years [6] and 1.5% among those aged 12–14 years [7]. Both pediatric CM and CDH are notably refractory. Previous studies have indicated that recovery tends to be gradual and incomplete, with high rates of symptom persistence or recurrence over time [8,9]. Although some patients improve early under clinical care, predictors of treatment responses remain poorly defined.

The chronification of headache is thought to involve several interacting mechanisms, including central sensitization [10] and impaired pain modulation [11], which may be promoted or sustained by comorbid conditions [12]. Persistent nociceptive inputs can contribute to persistent headaches and functional disability [13]. Consistent with this, children with primary headaches demonstrate multimodal sensory hypersensitivity, including lower mechanical pain thresholds, heightened electrical stimulation detection sensitivity, and increased olfactory sensitivity compared to their healthy peers, a profile that may contribute to further chronification [14]. In many pediatric cases, these processes accompany the transition of a pre-existing episodic headache disorder into a daily pattern, with the daily state and underlying neurobiological changes reinforcing each other. Shortening the duration of this daily phase may prevent neuroplastic changes prior to the establishment of refractory chronification. However, this early transition period in children remains insufficiently studied, and existing literature has focused largely on long-standing, established CDH or CM, leaving a critical knowledge gap regarding the acute transition itself. Although a recent study evaluated the initial clinical outcomes and biopsychosocial factors in youths with continuous headache [9,15], the interval between headache onset and specialist consultation has received little systematic attention. Prior work has documented substantial diagnostic delays in pediatric headache [16], and this interval has been shown to vary significantly with patient age [17]. However, whether its duration influences subsequent clinical outcomes remains largely unexamined. In this study, we examined pediatric and adolescent patients whose preexisting headaches had transformed into daily headaches, investigated the optimal timing for initiating specialist evaluations after daily headache onset, and assessed its impact on headache improvement.

## 2. Materials and Methods

### 2.1. Study Design and Participants

This retrospective observational study included children and adolescents who presented to our tertiary university hospital with daily headaches between September 2020 and May 2025 and were followed at our institution for at least 3 months. When clinically indicated, magnetic resonance imaging and laboratory tests were performed to exclude secondary headache etiology, and patients with secondary headache disorders were excluded. Daily headache was defined as a headache occurring every day, and underlying headache diagnoses were made according to the criteria of the International Classification of Headache Disorders, Third Edition [5]. As all patients had a preexisting primary headache disorder before the onset of daily headache, patients with new daily persistent headache were not included in this cohort.

The study was conducted in accordance with the Declaration of Helsinki and approved by the Institutional Review Board of Hyogo Medical University (approval number: 5388). Because of the retrospective nature of the study, the requirement for informed consent was waived. Instead, study information was made accessible on the institutional website to allow individuals the option to decline participation.

### 2.2. Clinical Assessment and Variables

Specialists performed all headache evaluations. At the initial consultation, guidance on headache management and lifestyle modifications was provided to all patients, which was reinforced at each subsequent visit. Whether to initiate pharmacological treatment was determined through shared decision-making with the patient and family. The specific agent was selected at the physician’s discretion, and efficacy was assessed after approximately 2 months. The collected data included sex, age, comorbid conditions, and consultation lag, defined as the interval between the date on which the headache first became daily and the date of the initial consultation at our institution. Prior to the initial consultation, patients and their caregivers completed a standardized questionnaire documenting the age at initial headache onset and headache frequency at the time of consultation; the date on which the headache became daily was further clarified through a direct interview at the initial consultation, defined as the earliest date from which a daily pattern was identified. The consultation lag was determined at the initial consultation based on the headache history obtained from the patient and caregiver.

The following comorbid conditions were evaluated: neurodevelopmental disorders, psychosocial factors, postural orthostatic tachycardia syndrome (POTS), and sleep disorders. Neurodevelopmental disorders were identified according to the criteria outlined in the Diagnostic and Statistical Manual of Mental Disorders, Fifth Edition [18], and included attention deficit/hyperactivity disorder and autism spectrum disorder. Psychosocial factors were not defined solely by self-reported stress. At the initial consultation, these factors were screened using patient questionnaires and a validated Japanese checklist for psychosomatic disorders [19]. Psychosocial comorbidities were defined as cases in which a targeted psychotherapeutic or counseling intervention directed at identified psychosocial issues, beyond the general guidance provided to all patients, was undertaken. POTS was suspected in patients with orthostatic intolerance. An active standing test was performed, and the diagnosis was made based on established pediatric criteria, defined as an increase in heart rate of ≥35 beats/min within 10 min of standing in the absence of orthostatic hypotension [20]. Sleep disorders were diagnosed according to the International Classification of Sleep Disorders (Third Edition) [21]. Information on sleep habits was obtained using questionnaires that assessed bedtime, sleep-onset latency, wake-up time, nocturnal awakening, and daytime sleepiness.

### 2.3. Outcome Measures

Clinical outcomes were evaluated at two follow-up windows: 3–4 months (primary outcome) and 6–7 months (secondary outcome) after the initial consultation. At each assessment, headache frequency was determined from the one-month period immediately preceding the visit and compared with the baseline. Improvement was defined as a ≥50% reduction in monthly headache days. Each endpoint was analyzed on a complete-case basis, and patients with missing outcome data at a given time point were excluded from the corresponding analysis.

### 2.4. Statistical Analysis

Statistical analyses were performed using SPSS software (version 31.0; IBM Corp., Armonk, NY, USA). Continuous variables are expressed as medians with interquartile ranges, and categorical variables are presented as frequencies and percentages. Baseline clinical characteristics and covariates were compared between patients with and without headache improvement at the 3–4- or 6–7-month endpoints using the Mann–Whitney U test for continuous variables and the chi-square test (or Fisher’s exact test when expected cell counts were small) for categorical variables. As a descriptive analysis, patients were also stratified into two age groups (4–10 and 11–16 years), and consultation lag and improvement rates at 3–4 months were compared between the groups using the same statistical approach. Comorbid conditions were compared individually between groups in a univariable analysis and were entered as a single composite variable (presence of any comorbidity) in multivariable models. Prophylactic agents (cyproheptadine, amitriptyline, and lomerizine) were combined into a single binary variable (administered vs. not) for multivariable modeling, because analyzing each agent individually would have increased the number of covariates relative to the number of outcome events, further reducing the already limited events-per-variable ratio in our models.

Multivariable logistic regression analyses were performed to identify the independent predictors of headache improvement. Before modeling, multicollinearity among the covariates was evaluated using the variance inflation factor, with values < 5 considered indicative of the absence of significant multicollinearity. In the initial multivariate model, consultation lag was entered as a continuous independent variable with simultaneous adjustment for predefined potential confounders, including age at initial consultation, sex, presence of comorbid conditions, and administration of prophylactic drugs. Given the relatively low number of improvement events relative to the number of covariates, Firth’s penalized logistic regression was additionally performed as a sensitivity analysis for the continuous and dichotomized consultation-lag models at both time points, using R (version 4.2.2; R Foundation for Statistical Computing, Vienna, Austria) and the logistf package (version 1.26.0), with confidence intervals calculated using the profile penalized log-likelihood method). As an additional sensitivity analysis, all multivariable models were repeated after restricting the cohort to patients with a migraine diagnosis.

Subsequently, to explore a clinically actionable threshold for the timing of intervention, receiver operating characteristic (ROC) curve analysis was performed using the 3–4-month primary outcome data. The optimal cutoff value for the consultation lag was identified by maximizing the Youden index, and the consultation lag was dichotomized at this threshold. A final multivariable logistic regression analysis was then performed using the dichotomized variable (>5 weeks vs. ≤5 weeks), with ≤5 weeks as the reference category, alongside the aforementioned covariates to evaluate its independent association with headache improvement at the 3–4-month and 6–7-month follow-ups. Adjusted odds ratios (aORs) and corresponding 95% confidence intervals (CIs) were calculated for each model. All statistical tests were two-tailed, and a *p*-value < 0.05 was considered statistically significant.

## 3. Results

**Figure 1:**
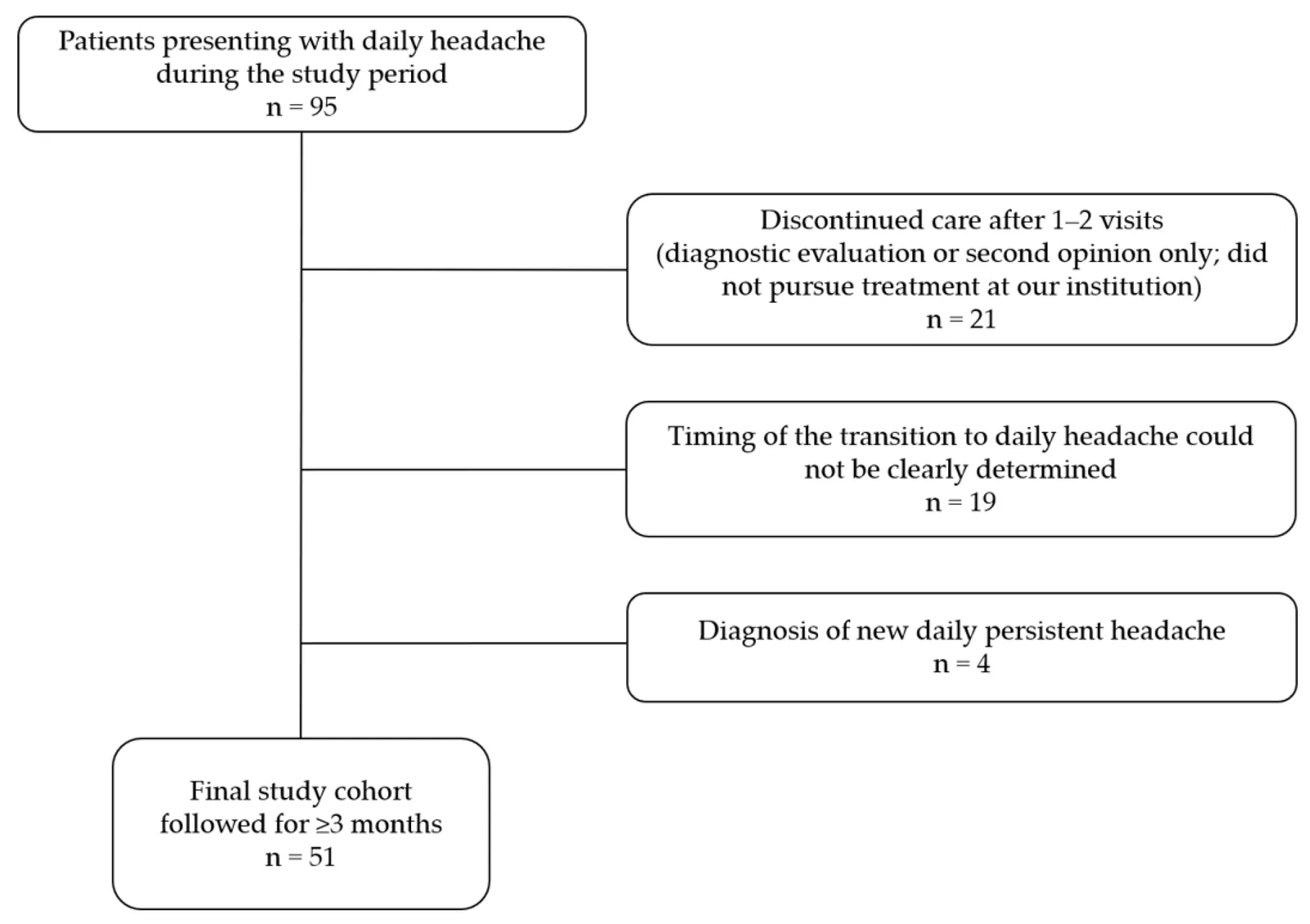
Flow diagram of patient screening and inclusion.

Of 95 patients who initially presented with daily headaches during the study period, 51 were included in the final study cohort after exclusions (Figure 1).

One patient was subsequently transferred to the psychiatric department and did not return for follow-up; thus, 50 patients had outcome data available at 6–7 months. Baseline and demographic characteristics of the 51 patients are presented in Table 1. The median age at initial consultation was 12 y and 11 months (range, 4 year and 3 months to 16 year and 11 months). Migraine was the most common primary headache subtype (45 patients, 88.2%), followed by tension-type headaches (4 patients, 7.8%) and other primary headaches (2 patients, 3.9%). The patient who was lost to follow-up before the 6–7 month assessment had a consultation lag of 32 weeks, was 13 years of age at the initial consultation, had psychosocial factors as the only recorded comorbidity, and had not achieved improvement at 3–4 months.

The two cases classified as other primary headaches could not be further categorized because the duration of individual headache attacks was insufficient to meet the criteria for a specific subtype, although their clinical features were consistent with those of migraine. Twenty-two patients (43.1%) were male, and 29 (56.9%) were female. The median consultation lag was 8 weeks (range, 1 to 72). Regarding comorbid conditions, 11 patients (21.6%) had none, 8 (15.7%) had neurodevelopmental disorders, 25 (49.0%) had psychosocial factors, 10 (19.6%) had POTS, and 19 (37.3%) had sleep disorders. No patient in this cohort met the criteria for medication-overuse headache. Prophylactic drugs, including cyproheptadine, amitriptyline, and lomerizine, were administered to 27 (52.9%) patients. Among these, cyproheptadine was used in 11 patients, amitriptyline in 5, and lomerizine hydrochloride in 13; some patients received more than one agent concurrently.

Headache improvement was observed in 21 of the 51 patients (41.2%) at 3–4 months, and in 30 of the 50 patients (60.0%) at 6–7 months. Table 2 compares the baseline characteristics of patients with and without improvement at each time point. At both time points, the improved group had a significantly lower median age at initial consultation (3–4 months: 11.5 vs. 13 years, *p* = 0.010; 6–7 months: 12 vs. 13 years, *p* = 0.029). To further explore the age-related pattern, we additionally compared consultation lag and improvement rates between two age groups (4–10 years, n = 11, and 11–16 years, n = 40). The younger age group showed a numerically shorter consultation lag (median, 4 vs. 11 weeks; *p* = 0.058) and a higher proportion of improvement at 3–4 months (63.6% vs. 35.0%; *p* = 0.165), consistent with the findings of the primary analysis; however, neither difference reached statistical significance, likely attributable to the small number of patients in the younger age group. The median consultation lag was 4 weeks in the improved group and 12 weeks in the non-improved group at both time points, and was significantly shorter in the improved group (*p* < 0.001 at 3–4 months; *p* = 0.017 at 6–7 months). Sex did not differ significantly between groups at either time point. Among the comorbid conditions analyzed individually, POTS was significantly associated with a lack of improvement at 3–4 months, although this association was no longer observed at 6–7 months (Table 2); no other comorbidities were significantly associated with improvement at either time point, and the administration of prophylactic drugs was likewise not significantly associated with improvement.

In the multivariate logistic regression models, all adjusted covariates showed a variance inflation factor below 2.0, indicating the absence of significant multicollinearity. In the model using the continuous consultation lag (Table 3), a longer consultation lag was significantly associated with a lower probability of improvement at 3–4 months (aOR, 0.875 per week; 95% CI, 0.783–0.979; *p* = 0.020), which indicated that each additional week of consultation lag was associated with an approximately 12% decrease in improvement; age, sex, presence of any comorbidity, and prophylactic drug administration were not significant. At 6–7 months, the continuous consultation lag was no longer significantly associated with improvement (aOR, 0.960 per week; 95% CI, 0.899–1.024; *p* = 0.213), and no other covariates reached statistical significance. The events-per-variable ratio was 4.2 at 3–4 months and 4.0 at 6–7 months, which were below conventional recommendations. Sensitivity analyses using Firth’s penalized logistic regression yielded materially consistent results at both time points (3–4 months: OR, 0.899; 95% CI, 0.800–0.978; *p* = 0.009; 6–7 months: OR, 0.969; 95% CI, 0.904–1.019; *p* = 0.239), supporting the robustness of the primary findings and the attenuation of the effect over time.

**Figure 2:**
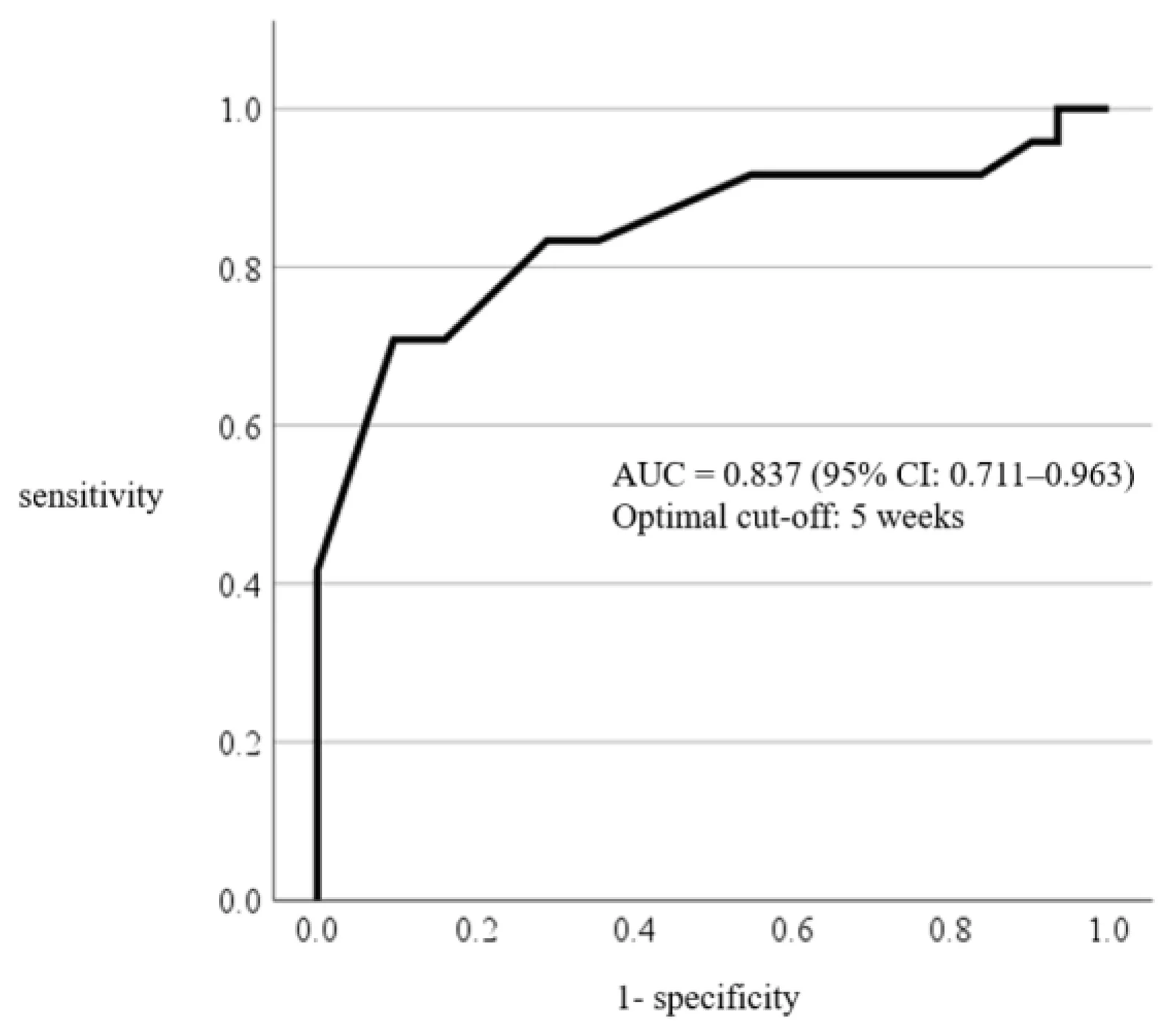
Receiver operating characteristic (ROC) curve for consultation lag and headache improvement at 3–4 months. Consultation lag was defined as the interval between the date on which the headache first became daily and the date of the initial consultation. The optimal cutoff of 5 weeks was identified by maximizing the Youden index, yielding a sensitivity of 0.714 and a specificity of 0.900 (AUC, 0.837; 95% CI, 0.711–0.963; *p* < 0.001). AUC, area under the curve; CI, confidence interval; ROC, receiver operating characteristic.

To identify a clinically actionable threshold, we performed ROC curve analysis for improvement at 3–4 months, which yielded an area under the curve of 0.837 (95% CI, 0.711–0.963; *p* < 0.001). The optimal cutoff, identified by maximizing the Youden index, was 5 weeks, with a sensitivity of 0.714 and a specificity of 0.900 (Figure 2).

In the final multivariable model using the dichotomized 5-week threshold (Table 4), patients with a consultation lag of >5 weeks had significantly lower odds of improvement at 3–4 months than those who presented within 5 weeks (aOR, 0.040; 95% CI, 0.007–0.238; *p* < 0.001); age, sex, presence of any comorbidity, and prophylactic drug administration were not independently associated with improvement. At 6–7 months, the dichotomized threshold was not significantly associated with improvement (aOR, 0.224; 95% CI, 0.046–1.097; *p* = 0.065), and no other covariates showed a significant independent association.

In a sensitivity analysis restricted to patients with migraine (n = 45 at 3–4 months; n = 44 at 6–7 months), results were materially consistent with those of the overall cohort at 3–4 months (continuous model: aOR, 0.884; 95% CI, 0.791–0.989; *p* = 0.031; dichotomized model: aOR, 0.014; 95% CI, 0.001–0.170; *p* < 0.001) and of the continuous model at 6–7 months (aOR, 0.965; 95% CI, 0.901–1.034; *p* = 0.309). For the dichotomized model at 6–7 months, the association reached statistical significance in the migraine-only subgroup (aOR, 0.119; 95% CI, 0.018–0.786; *p* = 0.027), in contrast to the non-significant finding in the overall cohort. Given the pronounced effect sizes observed in the dichotomized models, Firth’s penalized logistic regression was also applied. In the overall cohort, the point estimate was attenuated toward the null but remained highly significant at 3–4 months (OR, 0.065; 95% CI, 0.011–0.276; *p* < 0.001) and non-significant at 6–7 months (OR, 0.270; 95% CI, 0.057–1.078; *p* = 0.064), consistent with the original estimates. In the migraine-only subgroup, results showed a similar pattern of attenuation toward the null while remaining significant (3–4 months: OR, 0.033; 95% CI, 0.003–0.198; *p* < 0.001; 6–7 months: OR, 0.171; 95% CI, 0.026–0.783; *p* = 0.022), confirming that the significant association at 6–7 months in this subgroup was not an artifact of unpenalized maximum-likelihood estimation.

## 4. Discussion

To our knowledge, this study provides the first evidence that the pre-consultation interval is associated with short-term clinical outcomes in pediatric CDH. In the continuous model, a shorter consultation lag was associated with a higher probability of improvement at 3–4 months, and each additional week of consultation lag was associated with an approximately 12% decrease in the odds of improvement. This incremental relationship is consistent with the concept, established largely in adults, that the transition from episodic to chronic headache is driven by potentially modifiable processes and that earlier adequate management may limit chronification [22,23]. Although evidence in the pediatric population remains limited, a similar principle has been proposed for children: early and effective treatment of episodic headache may prevent its transformation into a chronic form [24]. As referral and management decisions benefit from a discrete threshold rather than a per-week estimate, we explored a cut-off using ROC analysis; the consultation lag that best separated improved from non-improved patients at 3–4 months was 5 weeks. Although the area under the curve of 0.837 suggests reasonable discriminative ability in the present dataset, it should be interpreted with considerable caution as it was derived from a single-center retrospective cohort of 51 patients, and the optimistic bias inherent in small-sample ROC analyses is well recognized. This threshold was derived and tested within the same cohort and should be considered exploratory. When this exploratory threshold was entered into the multivariable model, a consultation lag exceeding 5 weeks was associated with markedly lower odds of a 3–4-month improvement in this cohort. Although this association was adjusted for age, sex, comorbidity, and prophylactic drug use, it may be partly attributable to unmeasured factors—such as baseline headache severity, disability, treatment adherence, and healthcare accessibility—that could independently influence both the timing of consultation and the likelihood of improvement; this possibility of residual confounding is discussed further below.

The 5-week threshold warrants further mechanistic consideration. Central sensitization, a key driver of headache chronification, is thought to develop over weeks of sustained nociceptive input, progressively lowering the threshold for pain signaling and rendering headaches increasingly refractory to treatment [10]. Supporting this framework in the pediatric context, children with primary headaches have been shown to exhibit multimodal sensory hypersensitivity, encompassing lower mechanical pain thresholds, heightened electrical stimulation detection sensitivity, and increased olfactory sensitivity relative to their healthy peers, a profile hypothesized to contribute to further chronification [14]. Although the precise timeline over which these neuroplastic changes become entrenched remains uncertain, experimental and clinical evidence suggest that the window for reversing central sensitization narrows substantially once a chronic daily pattern is established [22]. The 5-week threshold identified here may, therefore, approximate the point at which such changes begin to solidify, although this interpretation is speculative and requires prospective validation. It should be emphasized that this cutoff was derived and tested within the same single-center dataset of modest size, without internal or external validation; it should be regarded as an exploratory hypothesis-generating finding rather than a definitive clinical benchmark. Notably, a Firth-penalized sensitivity analysis of this dichotomized model attenuated the point estimate toward the null while preserving statistical significance, consistent with the anticipated optimism of unpenalized estimates in the near-separated, small-sample setting. Furthermore, as our institution is a tertiary referral center, our cohort likely represents more severe or treatment-refractory cases than those encountered in general pediatric practice; moreover, patients who discontinued care after only diagnostic evaluation were not included in the outcome analysis, further concentrating our cohort toward those who pursued ongoing treatment. The generalizability of this threshold to less specialized settings therefore remains uncertain.

At the 6–7-month endpoint, neither the continuous interval nor the dichotomized 5-week threshold remained independently associated with improvement, and no other covariates reached significance. One interpretation is that the marked difference observed at 3–4 months attenuates over a longer course, as patients who present later eventually improve once they receive an adequate duration of specialized management and prophylaxis. Consistent with this, the proportion of patients with improvement increased from 21 of 51 (41.2%) at 3–4 months to 30 of 50 (60.0%) at 6–7 months, broadly in line with a large pediatric headache-care cohort in which headache frequency decreased in more than half of the follow-up intervals while patients remained in specialist care [25]. Over longer periods, most children with migraine ultimately follow a favorable course [26]. These observations suggest that the prognostic influence of consultation lag is greatest early and wanes as treatment continues. Nonetheless, the loss of significance at 6–7 months may partly reflect the limited statistical power of this modest sample rather than the true disappearance of the effect, particularly as the dichotomized estimate remained borderline. Six-month outcomes may also be shaped more by factors not captured in our models—such as treatment adherence and the degree of lifestyle modification—than by the initial interval; in that same cohort, clinical worsening was independently associated with chronic migraine, depressive symptoms, and greater baseline disability rather than with any single temporal factor [25]. Notably, the patient lost to follow-up before this time point had a long consultation lag, had not improved at 3–4 months, and was subsequently referred for psychiatric care, suggesting this loss was unlikely to be random with respect to prognosis and may have slightly inflated the 6–7-month improvement estimate; however, the impact of a single patient is likely small. Thus, the principal clinical value of a shorter interval may lie not in altering the ultimate 6-month prognosis but in an association with faster early improvement and a shorter period of disability during the first 3–4 months. A sensitivity analysis restricted to patients with migraine yielded consistent results at 3–4 months, supporting robustness across subtypes. The 6–7-month dichotomized association reached significance in this subgroup despite showing non-significance in the overall cohort; this discrepancy is likely sample-driven and should be interpreted cautiously. This finding was also confirmed using Firth’s penalized regression, indicating that it does not reflect an artifact of unpenalized estimation.

Among the comorbidities examined, POTS was an exception. Neurodevelopmental disorders, psychosocial factors, and sleep disorders showed no association with headache improvement at either 3–4 or 6–7 months, whereas none of the 10 patients with comorbid POTS showed improvement at 3–4 months, and five of the 10 patients showed improvement by 6–7 months. Therefore, in the short term, it was comorbid POTS specifically, rather than comorbidities in general, that distinguished a more resistant early course. These subgroup findings should be regarded as exploratory; the POTS group comprised only 10 patients, and the other subgroups (e.g., neurodevelopmental disorders) were similarly small and likely underpowered to detect modest effects. Because none of the 10 patients with comorbid POTS showed improvement at 3–4 months, complete separation precluded the stable estimation of logistic regression coefficients for POTS as a standalone predictor, which is a recognized limitation of standard maximum likelihood estimation in small samples with zero cell counts. Therefore, POTS-specific signals should be interpreted as descriptive rather than inferential. Nonetheless, the isolated POTS signal is consistent with prior reports that headache, particularly migraine, is highly prevalent among patients with POTS and may share pathophysiological mechanisms such as autonomic dysfunction [27] and impaired cerebral vascular regulation [28]; such mechanisms could contribute to a more persistent early course and a delayed, rather than absent, response to treatment in children with comorbid POTS. Whether targeted management of POTS itself, such as increased fluid and sodium intake, graded orthostatic training, or pharmacological intervention, can accelerate headache improvement in this subgroup remains an important question for future studies.

Beyond the consultation lag, none of the remaining baseline factors (age, sex, and prophylactic drug use) were independent predictors of improvement. Younger age was associated with improvement in the univariable comparisons but lost significance after adjustment, most likely because younger children tended to present sooner after headache onset, consistent with prior observations that parents of younger children and their referring physicians may respond more promptly to headache complaints [17]. A similar pattern was observed in a descriptive comparison across age groups, with the younger group showing a numerically shorter consultation lag and a higher proportion of early improvement; however, these differences did not reach statistical significance given the small stratum size. Therefore, the age effect observed in univariable analysis was likely confounded by the consultation lag rather than representing an independent prognostic factor. The lack of an independent effect of prophylactic drugs should be interpreted with caution for several reasons. First, the agents were chosen at the physician’s discretion and were generally reserved for more frequent or refractory headaches, introducing confounding by indication that would bias any observed effect toward the null. Second, all patients received lifestyle guidance regardless of drug use, making it difficult to isolate their pharmacological effects. Third, prophylactic agents were analyzed as a single binary variable rather than individually, as the sample size was insufficient to examine each agent separately. Given the mechanistic heterogeneity among cyproheptadine, amitriptyline, and lomerizine, this approach may have obscured differential treatment effects and reflects the same sample-size constraint underlying the low events-per-variable ratio discussed above. Fourth, the efficacy of preventive drugs in pediatric migraine is uncertain, given the high placebo response in randomized trials [29,30]. Overall, the timing of the presentation appeared to be the dominant modifiable correlate of early improvement.

### Limitations

This study had several limitations. The retrospective design precludes causal inference; the observed associations do not establish that presenting within 5 weeks directly caused subsequent improvement. Factors such as headache severity, disability, parental health-seeking behavior, and socioeconomic status may have influenced consultation timing and prognosis, but such information was not available in our retrospective dataset; this unmeasured confounding could not be excluded. Improvement of headache was defined solely by a ≥50% reduction in headache frequency. Headache intensity and disability were intended to be assessed during routine follow-up but were too incompletely recorded to permit quantitative analysis; thus, clinically meaningful changes not reflected in frequency alone may have been missed. As a tertiary pediatric headache clinic, our institution manages a selected population, and selection bias related to referral patterns and continuation of care cannot be excluded, thereby limiting its generalizability. Notably, 21 of 95 screened patients (22%) discontinued care after only one or two visits, having sought diagnostic evaluation or a second opinion rather than ongoing treatment at our institution; their exclusion may have further concentrated the analyzed cohort toward patients who chose to pursue continued treatment, potentially those with more persistent or treatment-motivated presentations. Although daily headache onset was recorded via a standardized pre-consultation questionnaire, confirmed at an interview, and corroborated by referral documentation when available, some recall-related imprecision could not be excluded. Such non-differential measurement error would generally attenuate associations and blur the precision of the ROC-derived cutoff, though it could not be quantified in this retrospective design. Consistent with this concern, 19 patients were excluded during screening because the timing of the transition to daily headache could not be determined, indicating that some residual imprecision in this exposure measure likely remains even within the analyzed cohort. Finally, this was a single-center study with a modest sample size, which had several downstream consequences. It precluded adequately powered subgroup analyses using specific prophylactic agents or individual comorbid conditions, and, for the same reason, we were unable to assess whether treatment choice varied according to headache severity, as standardized severity measures were not available. Moreover, prophylactic agents were selected at the discretion of the attending physician, rather than according to a predefined protocol. This limited sample size also contributed to a low events-per-variable ratio (4.0–4.2) in our multivariable models, below conventional recommendations; however, Firth penalized regression sensitivity analyses of the continuous consultation-lag models yielded materially consistent estimates at both time points. For the dichotomized models, Firth’s method attenuated the point estimates toward the null (e.g., 3–4 months: OR, 0.065 vs. the original aOR, 0.040) while preserving statistical significance, consistent with the expected optimism of unpenalized estimates in the small, near-separated sample; a similar pattern was observed in the migraine-only subgroup. For the same reason, the exploratory comparison across age groups was limited by the small number of patients in the younger age group (n = 11), and the resulting estimates, though numerically consistent with the main findings, did not reach statistical significance. Prospective multicenter studies with larger, well-defined cohorts are required to validate the 5-week threshold, establish causality, and optimize intervention timing for pediatric daily headaches.

## 5. Conclusions

The present study found that the interval between daily headache onset and initial specialist consultation was independently associated with short-term improvement in children with daily headaches, regardless of the comorbidity burden. Comorbid neurodevelopmental disorders, psychosocial factors, and sleep disorders were not associated with outcome, suggesting that in this cohort, early referral timing—rather than comorbidity burden—was associated with early improvement. Comorbid postural orthostatic tachycardia syndrome was the exception, being associated with a more resistant early course. An exploratory 5-week threshold was identified, beyond which early improvement was substantially less likely. As these findings are derived from a single tertiary pediatric headache center, they should be generalized to other clinical settings with caution. Prospective multicenter studies are needed to validate these findings and provide evidence-based referral guidelines for daily pediatric headaches. In the interim, these findings may support timely specialist referral when daily headache persists beyond several weeks, particularly to inform discussions between clinicians and families regarding the urgency of evaluation.

## Figures and Tables

**Table 1:** Baseline and demographic characteristics of the study population.

Total number of participants	51
Median age at initial consultation, years (range)	12 y11 month (4 y3 month–16 y11 month)
Duration from headache onset to daily headache, n (%)	
<1 year	17 (33.3)
1–2 years	16 (31.4)
2–5 years	14 (27.5)
≥5 years	4 (7.8)
Classification of primary headache	
migraine	45 (88.2)
tension-type headache	4 (7.8)
other primary headache	2 (3.9)
Sex, n (%)	
Male	22 (43.1)
Female	29 (56.9)
Consultation lag, week, median (range)	8 (1–72)
Comorbid conditions, n (%)	
None	11 (21.6)
Neurodevelopmental disorder	8 (15.7)
Psychosocial factors	25 (49.0)
POTS	10 (19.6)
Sleep disorder	19 (37.3)
Administration of prophylactic drugs, n (%)	
Yes	27 (52.9)
No	24 (47.1)

Data are presented as median (range) or n (%). Migraine includes chronic migraine and probable migraine. The two cases of other primary headache could not be further subclassified owing to insufficient attack duration, although their clinical features were consistent with migraine. Comorbid conditions are not mutually exclusive; patients may have more than one comorbidity. Abbreviation: POTS, postural orthostatic tachycardia syndrome.

**Table 2:** Comparison of baseline characteristics between patients with and without headache improvement at 3–4 and 6–7 months.

	3–4 Months			6–7 Months		
	Improved n = 21	Not Improved n = 30	*p*-Value	Improved n = 30	Not Improved n = 20	*p*-Value
Median age at initial consultation, years (range)	11.5 (4–14)	13(4–16)	0.010	12 (4–15)	13 (9–16)	0.029
Sex, n (%)						
Male	10	12	0.774	15	7	0.387
Female	11	18		15	13	
Consultation lag, week, median (range)	4 (1–36)	12 (4–72)	<0.001	4 (1–36)	12 (4–72)	0.017
Comorbid conditions						
Neurodevelopmental disorder						
Yes	4	4	0.702	5	3	1.000
No	17	26		25	17	
Psychosocial factors						
Yes	11	14	0.779	15	9	0.779
No	10	16		15	11	
POTS						
Yes	0	10	0.003	5	5	0.494
No	21	20		25	15	
Sleep disorder						
Yes	7	12	0.771	10	9	0.553
No	14	18		20	11	
Administration of prophylactic drugs						
Yes	10	17	0.578	16	10	1.000
No	11	13		14	10	

Data are presented as median (range) or n. Improvement was defined as a ≥50% reduction in monthly headache days from baseline. Age is expressed in years. *p*-values for continuous variables were calculated using the Mann–Whitney U test; *p*-values for categorical variables were calculated using the chi-square test or Fisher’s exact test, as appropriate. Comorbid conditions were analyzed individually in this table. Abbreviation: POTS, postural orthostatic tachycardia syndrome.

**Table 3:** Multivariable logistic regression analysis of factors associated with headache improvement.

	3–4 Months			6–7 Months		
Variables	aOR	95%CI	*p*-Value	aOR	95%CI	*p*-Value
Age	1.002	(0.975–1.029)	0.900	0.986	(0.957–1.016)	0.356
Sex	0.968	(0.243–3.863)	0.964	0.662	(0.187–2.349)	0.523
Consultation lag	0.875	(0.783–0.979)	0.020	0.960	(0.899–1.024)	0.213
Comorbid conditions	0.114	(0.011–1.170)	0.068	0.494	(0.064–3.835)	0.500
Prophylactic drugs	0.557	(0.134–2.324)	0.422	0.937	(0.261–3.370)	0.921

All covariates were entered simultaneously. Consultation lag: continuous (per week); Age: continuous (per year); Sex: binary (male vs. female); Comorbid conditions: binary (any comorbidity vs. none); Prophylactic drugs: binary (administered vs. not). Abbreviations: aOR, adjusted odds ratio; CI, confidence interval.

**Table 4:** Multivariable logistic regression analysis of factors associated with headache improvement, with consultation lag dichotomized at the 5-week threshold.

	3–4 Months			6–7 Months		
Variables	aOR	95%CI	*p*-Value	aOR	95%CI	*p*-Value
Age	1.006	(0.977–1.036)	0.679	0.988	(0.961–1.017)	0.426
Sex	0.619	(0.129–2.981)	0.550	0.549	(0.149–2.016)	0.366
Consultation lag (>5 weeks vs. ≤5 weeks)	0.040	(0.007–0.238)	<0.001	0.224	(0.046–1.097)	0.065
Comorbid conditions	0.109	(0.008–1.421)	0.091	0.574	(0.067–4.918)	0.612
Prophylactic drugs	0.577	(0.116–2.875)	0.502	1.105	(0.297–4.120)	0.881

All covariates were entered simultaneously. Consultation lag: binary (>5 weeks vs. ≤5 weeks); Age: continuous (per year); Sex: binary (male vs. female); Comorbid conditions: binary (any comorbidity vs. none); Prophylactic drugs: binary (administered vs. not). Abbreviations: aOR, adjusted odds ratio; CI, confidence interval.

## Data Availability

The original contributions presented in this study are included in the article. Further inquiries can be directed to the corresponding author.

## Abbreviations

The following abbreviations are used in this manuscript:

CM	Chronic migraine
CDH	Chronic daily headache
POTS	Postural orthostatic tachycardia syndrome
CI	Confidence interval
aOR	Adjusted odds ratio
